# Entropy-driven online open circuit voltage identification for precise state estimation in lithium-ion batteries

**DOI:** 10.1016/j.isci.2025.113290

**Published:** 2025-08-06

**Authors:** Zhengyang Li, Cheng Chen, Ruixin Yang, Hailong Li, Rui Xiong

**Affiliations:** 1 Department of Vehicle Engineering, School of Mechanical Engineering, Beijing Institute of Technology, Beijing 100081, China; 2 School of Business, Society and Engineering, Mälardalen University, 72123 Västerås, Sweden

**Keywords:** Thermodynamics, Electrochemistry, Energy engineering

## Abstract

The open circuit voltage (OCV)—state of charge (SOC) curve of lithium-ion batteries is affected by battery inconsistency and degradation. Compared to lab methods, which are time-consuming, using operation data of electric vehicles (EVs) to identify OCV-SOC curve online attracts increasing attention. Considering that many operating conditions of EVs cannot sufficiently excite the dynamic voltage response of battery, leading to significant uncertainty in identification results, the Shannon entropy of measured signal and terminal voltage error calculated by the identified parameters are used to assess the accuracy of the identified OCV in this work. Then the identified OCV is used to interpolate the start point of ampere-hour counting in the constructed OCV-SOC segment, to guarantee the accuracy of SOC. Validation results show that the maximum deviation of the online constructed OCV-SOC curve is below 22 mV. When applied to SOC estimation, an error of less than 2.2% can be achieved.

## Highlights

• Reliable method for online construction of OCV-SOC curve is proposed
• Shannon entropy and voltage error assess parameter identification accuracy
• Accurate OCV is used to obtain the start point of ampere-hour counting
• Proposed method is verified under various operating conditions

## Graphical abstract

**Figure:**
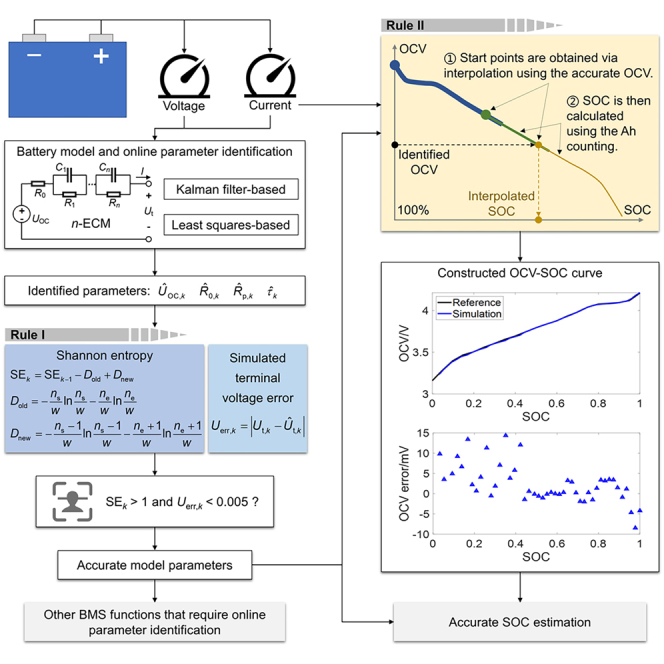

## Introduction

The development of electric vehicles (EVs) is a key measure for building a low-carbon transportation system and promoting sustainable development.^1^^,^^2^ Currently, lithium-ion batteries (LIBs) are the preferred energy storage solution for EVs due to their high energy density, long cycle life, and low self-discharge rate.^3^^,^^4^ To ensure the safe and efficient operation of LIBs, a battery management system (BMS) is essential.^5^ Accurate estimation of battery state is an important function of BMSs and it heavily relies on the open circuit voltage (OCV)-state of charge (SOC) curve.^6^^,^^7^^,^^8^

The OCV-SOC curve can be obtained through lab tests, including incremental OCV tests,^9^ low current tests,^10^^,^^11^ and hybrid pulse power characteristic tests,^12^ all of which are time-consuming due to the inconsistency of different battery cells. Moreover, with the increase of battery lifespan, obtaining the OCV-SOC curve in the whole life of the battery requires even longer time.

In addition to the direct measurement, other methods for the construction of OCV-SOC curve were proposed, which can be classified into three categories: data driven methods, which require a lot of experimental test data or vehicle operation data to train models or mine information, electrode open circuit potential (OCP) matching methods, which is based on the principle that the OCV can be constructed by scaling and shifting the OCP curves of the positive and negative electrodes,^13^ and online parameter identification-based methods, which treats OCV as the parameter to be identified in the equivalent circuit model (ECM). A comparison of different methods is shown in Table 1. In general, both the data driven and OCP matching methods have a requirement of large amount of data or high-computational complexity, which limits their online applications. In contrast, the online parameter identification-based methods, with their moderate computational cost and memory usage, are more suitable for real-time computation in embedded systems. Various online identification techniques for ECM parameters have been proposed and have demonstrated excellent performance under dynamic operating conditions,^14^^,^^15^^,^^16^^,^^17^ enabling OCV to be accurately identified in real time. Among them, the extended Kalman filter (EKF) and forgetting factor recursive least square (FFRLS) are widely acknowledged as representative approaches. However, online parameter identification does not always yield accurate results under real operating conditions of EVs. Zhu et al.^18^ analyzed the limitation of FFRLS and pointed out that under steady-current conditions, the identification results may be unreliable. Specifically, when the regression vector (composed of current and voltage) fails to excite the entire information space, the unexcited subspace is discounted continuously without receiving new informative data. As a result, the eigenvalues of the information matrix that correspond to the unexcited subspace tend to zero, and the covariance matrix goes to infinity. This leads to extremely noisy estimates that cannot track the actual changes of parameters.^19^ The EKF faces a similar challenge: when the current excitation is insufficient, such as constant-current charging or steady low-current parking, the dynamic response characteristic of battery voltage cannot be fully excited, making it difficult to accurately determine multiple model parameters simultaneously. Using incorrect identification results will directly affect the OCV-SOC curve construction process and may also cause BMS malfunction. Therefore, it is essential to take all possible scenarios into consideration to ensure functional safety and system stability. However, research on operating condition identification techniques and evaluation methods for parameter identification results remains relatively scarce. To the best of our knowledge, variance is commonly used by practitioners to assess current fluctuations. Moreover, as variance can be computed iteratively,^20^ it is well-suited for online applications. However, we found that during the transition of current from one steady state to another, or in the presence of a few outliers, the variance may increase sharply, which can lead to misjudgement.

**Table 1:** A summary of OCV-SOC curve construction methods

Category	Reference	Method	Accuracy	Pros and cons
Data driven	Chen et al.^21^	Segment scaling and movement	The SOC estimation error is less than 3.0%.	High accuracy, high computational complexity; requirement of large amount of experimental data or vehicle operation data.
	Zhou et al.^22^	Autoencoder, deep neural network	The average root-mean-square error (RMSE) of the OCV-capacity curve is less than 3 mAh.	
OCP matching	Cui et al.^23^	Particle swarm optimization	The RMSE in OCV construction is less than 20 mV.	Ability to reveal the mechanism of battery aging; high computational complexity; need of destructive tests of LIBs; parameter optimization introduces computational pressure.
	Guo et al.^24^	Hybrid particle swarm optimization–genetic algorithm, long short-term memory recurrent neural network	The mean absolute error (MAE) in OCV construction is less than 20 mV.	
Online parameter identification-based	Zhang et al.^25^	Extended Kalman filter (EKF)	Both MAE and RMSE of SOC estimation is below 1%.	Low computational complexity; high uncertainty of online parameter identification results.
	Chen et al.^26^	Forgetting factor recursive least square (FFRLS)	Maximum error of SOC estimate is less than 3%.	
	Wang et al.^27^	Fixed-memory recursive least squares–extended Kalman filtering–Kalman filtering	The maximum SOC estimation error is only 0.05% at 25°C	

On the other hand, under dynamic conditions with sufficient current excitation, the identified parameters may fluctuate due to the structure of ECM or the characteristics of the identification algorithm. By selecting the most appropriate parameters from these results, the accuracy of the constructed OCV-SOC curve can be further enhanced.

In order to solve the problem, a rule is proposed in this work to evaluate the accuracy of the identified parameters, including OCV. The ECM and EKF are employed to online identify OCV during vehicle operation. At each time step, the Shannon entropy (SE) of the measured signal and the terminal voltage error calculated by identified parameters together determine whether the identified OCV is accurate enough to construct the OCV-SOC curve. In addition, in order to obtain accurate SOC corresponding to the OCV, another rule is proposed. The ampere-hour (Ah) counting is used to calculate SOC during vehicle operation, with the start point determined by interpolating the identified OCV within the constructed OCV-SOC segment to avoid the accumulation of errors in Ah counting. These two rules run in real time, ensuring the accuracy of online construction of the OCV-SOC curve. Moreover, the proposed rules can not only provide support for most model-based SOC estimation methods but also contribute to enhance the performance and stability of other BMS functions that rely on online parameter identification.

**Figure 1:**
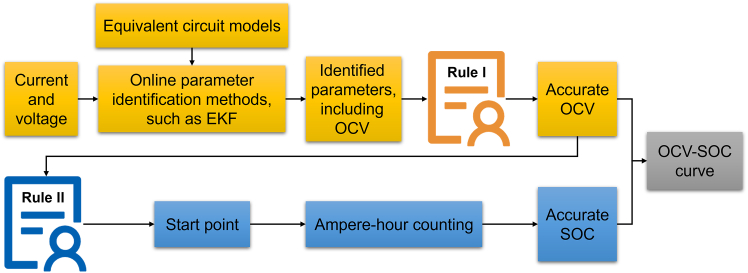
Overview of the proposed OCV-SOC curve online construction method The rule I and rule II are added to the online parameter identification and Ah counting to ensure the accuracy of OCV and SOC, respectively.

An overview of the proposed method is shown in Figure 1, where rule I and rule II are added to the online parameter identification and Ah counting to ensure the accuracy of OCV and SOC, respectively. Further details are provided in STAR Methods.

## Results and discussion

### The effectiveness of rule I

**Figure 2:**
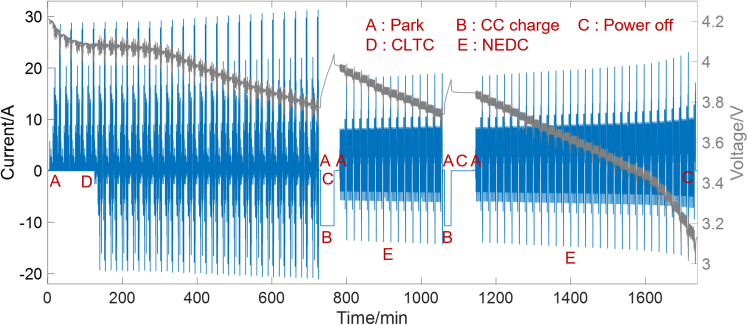
Current and voltage curve of the combined condition

**Figure 3:**
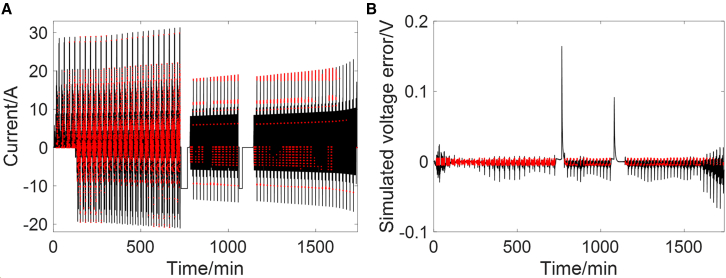
Evaluation results for the combined condition of rule I (A) Current, where the red points represent the moments when rule I is met. (B) Simulated terminal voltage error, where the red points represent the moments when rule I is met.

In order to simulate the complex real-world operating conditions of EVs, various common operating scenarios are combined together, as shown in Figure 2. China light-duty vehicle test cycle (CLTC) and new European driving cycle (NEDC) are used to represent the normal driving condition, constant low-current discharge simulates the parking condition, and constant current charge reflects the charging condition. The EKF is implemented to the combined condition and parameter identification at each time step is evaluated using rule I. The moments that meet rule I are shown in Figure 3. It can be seen from Figure 3A that the constant current charging and constant low-current parking conditions can be quickly identified and excluded, ensuring that only data points from dynamic conditions are retained. Figure 3B shows that under dynamic conditions, the identified parameters exhibit some fluctuations, particularly in low SOC range where model accuracy declines. Rule I enables the selection of the most accurate identified results which are then used to construct the OCV-SOC curve and support rule II.

**Figure 4:**
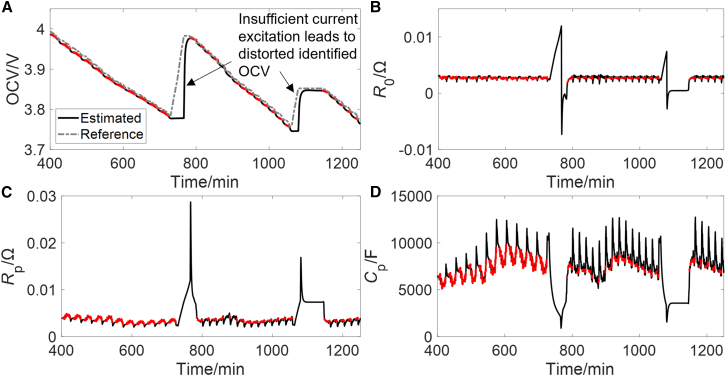
Identified ECM parameters of the combined condition The identified parameters are OCV (A), *R*_0_ (B), *R*_p_ (C), and *C*_p_ (D), where the red points represent the moments when rule I is met.

The identification results of four ECM parameters are shown in Figure 4. It can be seen that insufficient current excitation leads to distorted identified results, although the influence of historical data does not cause it to diverge quickly. Rule I ensures that these divergent parameters are not used. Some practitioners assess the validity of the identification results solely based on the simulated terminal voltage error, which may lead to some issues. Take the constant current charging condition as an example for analysis. Since the EKF performs feedback correction based on the measured terminal voltage, the simulated terminal voltage error remains small (less than 5 mV), as shown in Figure 3B. However, the current excitation is very insufficient, making it difficult to distinguish the contributions of four model parameters to the terminal voltage, which increases the likelihood of the algorithm converging to an incorrect parameter combination. As shown in Figure 4, the identified OCV remains nearly constant, while the increase in terminal voltage is attributed to an increase in internal resistance. A similar situation can also occur under steady discharge conditions. Therefore, relying solely on the simulated terminal voltage error is inadequate.

**Figure 5:**
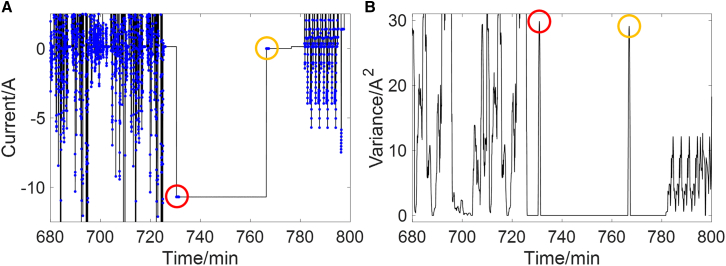
Evaluation results for the combined condition of variance (A) Current, where the blue points represent the moments when the variance exceeds the threshold. (B) Variance of current.

For further comparative analysis, the variance of the current within the sliding window (SD) is calculated to evaluate the combined condition in real time. The results are shown in Figure 5, where the blue points represent the moments when the variance exceeds the set threshold (0.1). It can be observed that during transitions when the current changes from one steady current condition to another, the variance increases significantly, leading to the inclusion of many points. However, despite the big change in current magnitude, this fluctuation occurs only once and is insufficient to ensure accurate parameter identification. Although increasing the variance threshold can mitigate this issue, it comes at the cost of discarding a considerable number of valid data points from dynamic operating conditions. And it is difficult to achieve a balance between them. In contrast, rule I effectively addresses this issue. Although the variance is large, the current only falls within two subintervals, the SE remains small and rule I correctly excludes these points from use. Based on aforementioned analysis, it can be inferred that even when the current sensor failure causes outliers or a high-power onboard electrical device is suddenly activated, leading to an instantaneous current change, the proposed method can still maintain high robustness. In summary, the proposed method shows advantages in evaluating online parameter identification, which is beneficial to ensuring the functional safety of BMS.

**Figure 6:**
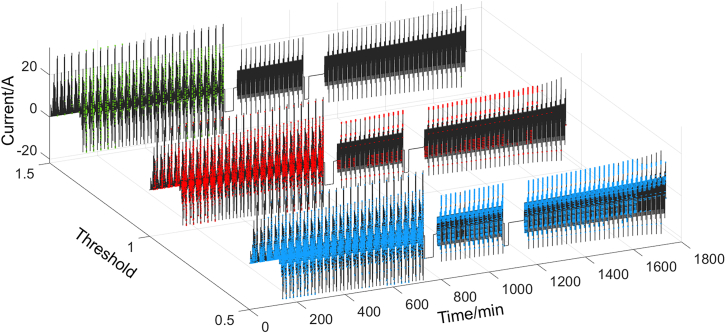
Evaluation results under the combined condition with SE thresholds of 0.5, 1, and 1.5, where the green, red, and blue points represent the moments when rule I is met

The threshold for SE is primarily determined by the distribution of current signal and is less affected by battery type or environmental condition. Figure 6 presents the evaluation results under the combined condition with SE thresholds of 0.5, 1, and 1.5. It can be seen that a threshold of 0.5 fails to effectively exclude transition processes in which the current falls into only two subintervals, while a threshold of 1.5 excludes too many points. Taking both resolution and data retention into account, a threshold of 1 achieves a desirable balance. In contrast, *U*_err_ is more significantly affected by factors, such as battery type and environmental condition. The applicability of ECMs varies across different battery types: while the modeling accuracy is generally high for nickel cobalt manganese (NCM) batteries, it may be lower for lithium iron phosphate (LFP) batteries. Additionally, the modeling accuracy of individual cells is typically much higher than that of battery packs. Under low-temperature conditions, modeling accuracy may also deteriorate considerably. Therefore, the threshold for *U*_err_ should be flexibly determined based on the specific application scenario. In this work, for a single NCM cell operating at room temperature, Figure 3B shows that most *U*_err_ is below 5 mV, which is sufficient for constructing the OCV-SOC curve. Accordingly, a threshold of 5 mV is adopted. For other scenarios, this value should be adjusted appropriately according to the parameter identification results and accuracy requirements.

**Figure 7:**
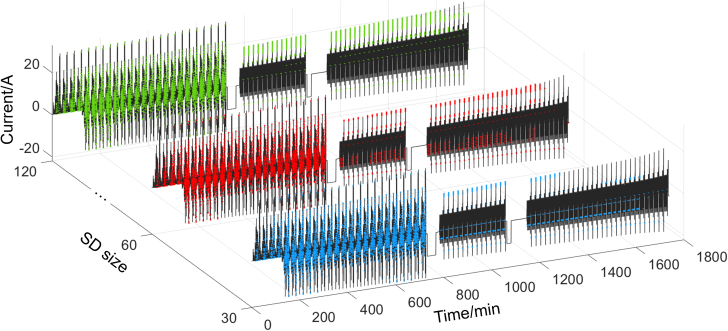
Evaluation results under the combined condition with SD sizes of 30, 60, and 120, where the green, red, and blue points represent the moments when rule I is met

The size of SD reflects the duration of operating condition being evaluated. A comparison of SD sizes of 30, 60, and 120 is presented in Figure 7. It can be seen that the constant current stages and the transition processes in between are all effectively excluded. The maximum SOC differences between adjacent selected points under dynamic conditions are 0.0148, 0.0207, and 0.0096, respectively, indicating that the number of selected points is all sufficient to support the modeling requirements of the OCV-SOC curve. Therefore, the method is not particularly sensitive to the size of SD.

### The effectiveness of rule II

**Figure 8:**
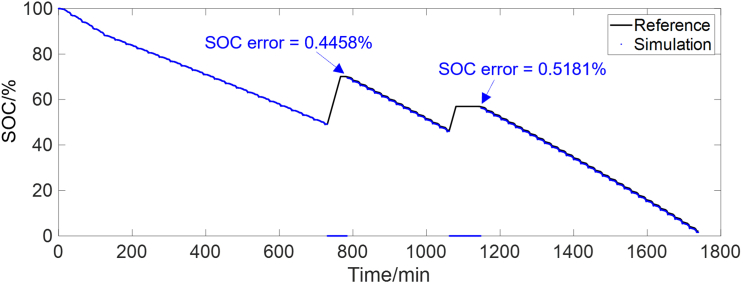
SOC calculation results by rule II Once the rule II is satisfied, the accurate SOC is retrieved again, with an error of less than 1%.

In rule II, the SOC is calibrated periodically, to prevent error accumulation of Ah counting. To verify the effectiveness of rule II, in the combined condition of Figure 2, the SOC is reset to zero when the battery starts constant current charging. The SOC calculation results by rule II are shown in Figure 8. It can be seen that once the rule II is satisfied, the accurate SOC is retrieved again, with an error of less than 1%. Then the Ah counting is used to calculate the subsequent SOC. When applying this strategy to EVs, the following aspects can be considered:

(1) During the application, multiple OCV points, such as 2 to 3 points with specific SOC intervals, can be used to calculate or verify the SOC. This will further enhance the reliability of the proposed method.
(2) Advanced interpolation techniques, such as spline interpolation, can be used to achieve higher accuracy. Moreover, the EKF can also be used to correct the SOC within the constructed OCV-SOC segment.
(3) This method is applicable only in regions where the OCV-SOC curve is steep. For LIBs with a flat OCV-SOC curve, such as LFP batteries, additional constraints are required. For example, the activation and deactivation of the associated strategies can be controlled by monitoring the magnitude of OCV variations.

### OCV-SOC curve construction results

**Figure 9:**
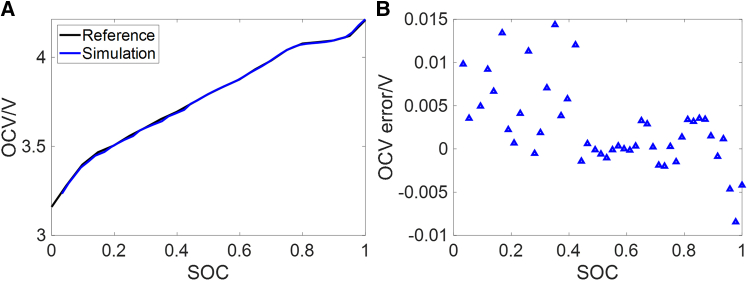
OCV-SOC curve construction results of the combined condition (A) Constructed OCV-SOC curve and reference curve. (B) Error of the constructed OCV-SOC curve.

Through the combined application of rule I and rule II, the OCV-SOC curve constructed under the combined condition is presented in Figure 9. It can be seen that the maximum absolute error (MaxAE) is below 15 mV, while the root-mean-square error (RMSE) and mean absolute error (MAE) are 5.3 mV and 3.6 mV, respectively. These results demonstrate that the proposed method can effectively construct the OCV-SOC curve during vehicle operation. Additionally, since the SOC interval of the construction points is limited to 2%–4%, and the accuracy of the identified OCV tends to deteriorate in the low SOC range, the final construction only reaches 3.30% SOC. In practice, the SOC range below 10% is seldom used in EVs,^28^ and some types of LIBs become increasingly difficult to model accurately in this range. Therefore, the length of the constructed OCV-SOC curve is sufficient for general applications.

The introduction of a sliding window and multiple judgments will increase memory usage, so we record the memory consumption of all variables and flags required by the algorithm. When the double data type is used, the total memory consumption is approximately 2,232 bytes, which can be met by most embedded controllers used in EVs.

### Application in SOC estimation

**Figure 10:**
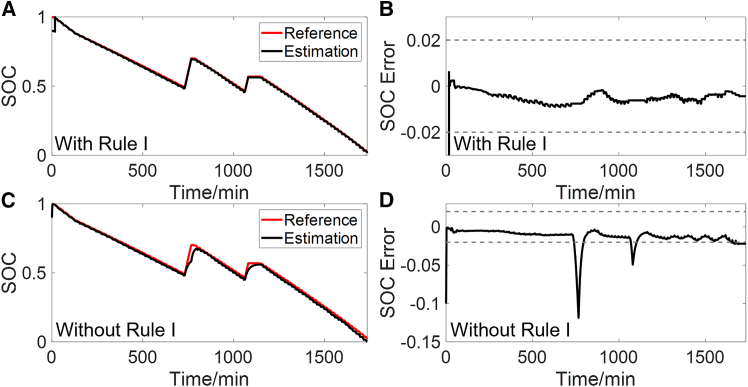
SOC estimation results using the constructed OCV-SOC curve under the combined condition with and without rule I (A and B) Estimated SOC (A) and its error (B), with rule I applied. (C and D) Estimated SOC (C) and its error (D), without rule I applied.

This section further illustrates the application potential of the proposed method by employing a commonly used SOC estimation method based on the Thevenin model and EKF as an example, whose calculation process can be seen in Table S1.^29^ An initial SOC error of 10% is set. Additionally, the EKF is disabled when the SOC is below 5%, and Ah counting is used instead. The SOC estimation results using the constructed OCV-SOC curve under the combined condition are presented in Figure 10 and the error statistics are shown in Table 2. In Figures 10A and 10B, the EKF is disabled at times when rule I is not met. It can be seen that the SOC estimation achieves high accuracy, with a MaxAE of less than 1% after the initial error is corrected. The RMSE and MAE are 0.0113 and 0.0059, respectively.

**Table 2:** Error statistics of SOC estimation

Condition	RMSE	MAE
Combined condition	0.0113	0.0059
CLTC	0.0113	0.0063
NEDC	0.0157	0.0090
WLTC	0.0141	0.0099

It is worth noting that SOC estimation methods based on ECMs are highly sensitive to the accuracy of model parameters. The proposed rule I effectively prevents SOC estimation in the case of inaccurate parameter identification. As shown in Figures 10C and 10D, when rule I is not applied and the EKF remains continuously active, the SOC estimation results deteriorate significantly under some operating conditions, with the MaxAE exceeding 10%. These results confirm that rule I plays a crucial role in improving the functional safety of SOC estimation under complex conditions.

**Figure 11:**
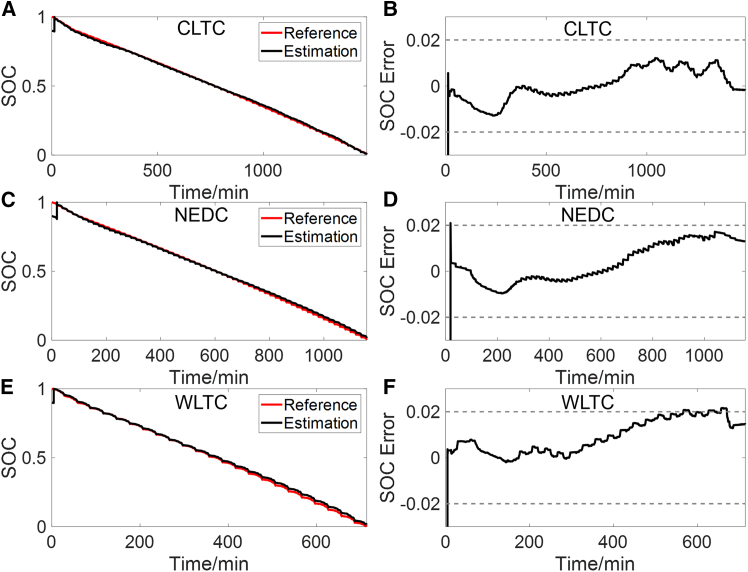
SOC estimation results using the constructed OCV-SOC curve under different dynamic operating conditions (A and B) Estimated SOC (A) and its error (B) under CLTC. (C and D) Estimated SOC (C) and its error (D) under NEDC. (E and F) Estimated SOC (E) and its error (F) under WLTC.

The SOC estimation results under CLTC, NEDC, and world light vehicle test cycle (WLTC) are shown in Figure 11, with corresponding error statistics provided in Table 2. After the initial error is corrected, the MaxAE is below 2.2% in all cases. The averages of RMSE and MAE are 0.0137 and 0.0084, respectively. This further demonstrates the accuracy of the constructed OCV-SOC curve.

### OCV-SOC curve construction results of aged battery

**Figure 12:**
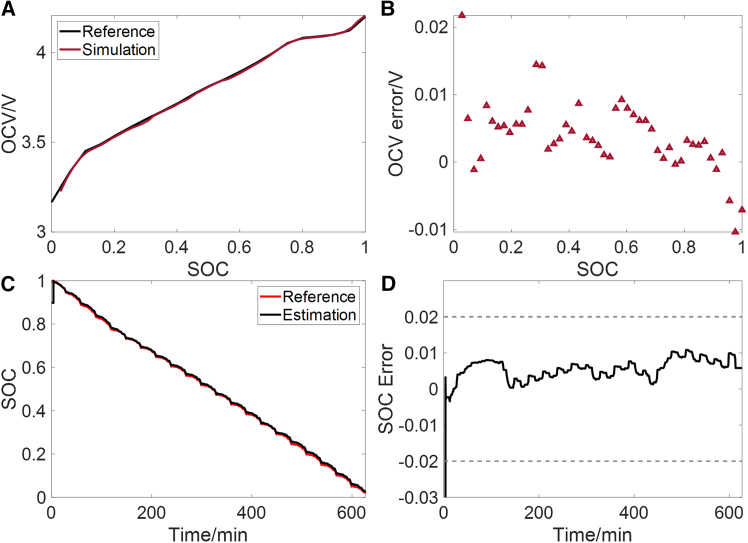
OCV-SOC curve construction results after battery aging and the corresponding SOC estimation results using the constructed curve (A and B) Constructed OCV-SOC curve of aged battery under the combined condition (A) and its error (B). (C and D) Estimated SOC (C) and its error (D) under WLTC using the constructed OCV-SOC curve.

In this section, a LIB of the same type, with a state of health (SOH) of 92.9% after 800 charge-discharge cycles, is used for validation. Under the same combined condition illustrated in Figure 2, the constructed OCV-SOC curve is shown in Figures 12A and 12B. The MaxAE is found to be below 22 mV, while the RMSE and MAE are 6.5 mV and 5.0 mV respectively. These results indicate that the proposed method can still construct an accurate OCV-SOC curve after the battery has aged. Based on the constructed OCV-SOC curve, the SOC estimation results under WLTC are shown in Figures 12C and 12D. Accurate SOC estimation is achieved, with a MaxAE of less than 2% after the initial error is corrected. The RMSE and MAE are 0.0098 and 0.0062, respectively.

## Discussion

In practical applications, this method should be appropriately adapted according to the specific application scenario. The following issues can be considered.

**Figure 13:**
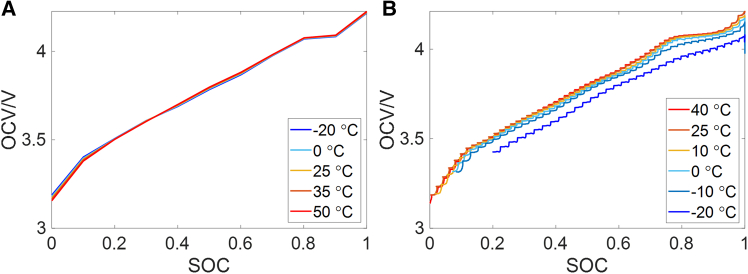
OCV-SOC curves measured and identified at different temperatures (A) OCV measured at different temperatures. (B) OCV identified from NEDC at different temperatures.
(2) The proposed method can be effectively extended to the cloud-BMS,^31^ serving as a candidate solution for mining and integrating information from real-vehicle big data.
(3) The proposed method can also be applied to the construction of OCV-state of energy (SOE) curves. However, since the estimation of SOE is significantly affected by heat generation of LIBs, the applicable temperature range requires further consideration and potential adjustment.

(1) The dynamic characteristics inside the battery vary significantly at different temperatures, which may affect the accuracy of battery model and online parameter identification. At low temperatures, even with sufficient current excitation, the identified OCV may still deviate significantly from the true value.^30^ For the LIB tested in this work, Figure 13 illustrates the measured OCVs and those identified from NEDC at different temperatures. It can be seen that the OCV identification error increases markedly at low temperatures, which will significantly affect the performance of the proposed method. For this type of LIB, 10°C can be regarded as a critical threshold. Consequently, in winter or high-altitude environments, the proposed method needs to be applied in conjunction with the thermal management system of EVs.

### Conclusion

This work provides a feasible solution for the online construction of OCV-SOC curve, which can reduce the offline experiments of LIBs. The EKF and Ah counting are used to identify OCV and calculate SOC. To ensure the accuracy of OCV and SOC, two rules are proposed. Rule I uses SE and simulated terminal voltage error to evaluate the accuracy of the identified OCV. The validated OCV is utilized by rule II to determine the start point of Ah counting, to avoid the accumulation of errors in Ah counting. Experiments are conducted to validate each component of the construction process and the main conclusions are summarized as follows.

(1) Under the combined condition, rule I effectively excludes scenarios where the accuracy of identified parameters cannot be guaranteed, such as steady current charging and parking conditions. This prevents inaccurate identified OCV from being used to construct the OCV-SOC curve. Additionally, the error of the start SOC points for Ah counting obtained through rule II is less than 1%. Ultimately, the MaxAE of the constructed OCV-SOC curve is less than 15 mV, with RMSE and MAE being 5.3 mV and 3.6 mV, respectively.
(2) SOC estimation based on ECM and EKF is conducted using the constructed OCV-SOC curve, and EKF is enabled only when rule I is met. After the initial error is corrected, the MaxAE under CLTC, NEDC, WLTC, and combined condition is less than 2.2%.
(3) The method is validated using an aged battery with an SOH of 92.9%. The MaxAE of the constructed OCV-SOC curve is less than 22 mV, with RMSE and MAE being 6.5 mV and 5.0 mV, respectively.

### Limitations of the study

The effectiveness of the proposed method largely depends on the accuracy of the battery model and online parameter identification, as well as the slope of the OCV-SOC curve. Consequently, the complex dynamic characteristics of LIBs at low temperatures or within low SOC ranges and the flat OCV-SOC profile of LFP batteries bring significant limitations to the method. Looking forward, we will incorporate more accurate battery models and advanced online parameter identification algorithms, while exploring more flexible calibration strategies to enhance the adaptability and robustness of the proposed method.

## Resource availability

### Lead contact

Requests for further information and resources should be directed to and will be fulfilled by the lead contact, Rui Xiong (rxiong@bit.edu.cn).

### Materials availability

This study did not generate new unique reagents.

### Data and code availability

• The data reported in this paper will be shared by the lead contact upon request.
• The key code used in this paper is available online at https://github.com/Wandering1113/OCV_online_construction.
• Any additional information required to reanalyze the data reported in this paper is available from the lead contact upon request.

## Acknowledgments

This work was supported by the 10.13039/501100001809National Natural Science Foundation of China (grant no. 52307233).

## Author contributions

Z.L., writing – original draft, methodology, and conceptualization; C.C., writing – review and editing, validation, and supervision; R.Y., investigation and conceptualization; H.L., writing – review and editing, supervision, conceptualization; R.X., writing – review and editing, methodology, supervision, and conceptualization.

## Declaration of interests

The authors declare no competing interests.

## STAR★Methods

### Key resources table

**Table:** 

REAGENT or RESOURCE	SOURCE	IDENTIFIER
**Software and algorithms**		

Python 3.9	Python Software Foundation	https://www.python.org
Key code	This paper	https://github.com/Wandering1113/OCV_online_construction

### Experimental model and study participant details

Tests are performed for an NCM LIB to validate the proposed method, which specifications are shown in Table S2. The experimental setup is shown in Figure S1. A thermal chamber is used to provide the required environmental temperature for battery experiments, with a temperature control accuracy of ±2°C. A battery test system is utilized to apply operating protocols to the battery and transmit information such as current, voltage, and temperature to a PC. The measurement accuracy of current, voltage and temperature is ±0.05%, ±0.05% and ±1°C respectively.

The incremental OCV test is conducted to obtain the OCV at different SOCs, with SOC intervals of 5%. Considering that the hysteresis effect of ternary batteries is not obvious, the OCV mentioned in this work corresponds to the discharge process. Additionally, various dynamic operating condition tests are carried out, including CLTC, NEDC, and WLTC. In order to simulate the complex real-world operating conditions of EVs, various common operating scenarios are combined together, as shown in Figure 2. CLTC and NEDC are used to represent the normal driving condition, constant low-current discharge simulates the parking condition, and constant current charge reflects the charging condition. Unless otherwise specified, the experiments discussed in this work are conducted at 25°C.

### Method details

#### ECM and online parameter identification

To balance accuracy and computational complexity, the Thevenin model is used in this work to characterize the dynamic response of battery voltage, which structure is shown in Figure S2. The OCV is represented by a voltage source (*U*_OC_). The ohmic resistance (*R*_0_) stands for the sum of the resistances of current collectors, electrodes, and electrolyte. The parallel RC network (*R*_p_ and *C*_p_) represents the polarization effect of the battery. *U*_t_ is the terminal voltage, and *I* is the current, with discharge being positive and charge being negative.

According to Kirchhoff’s law, the model expression is as follows:

(Equation 1) $$\left\{ \begin{array}{l} U_{t}\left(t\right)=U_{\text{OC}}\left(t\right)-I\left(t\right)R_{0}-U_{p}\left(t\right)\\ \frac{dU_{p}\left(t\right)}{dt}=-\frac{U_{p}\left(t\right)}{R_{p}C_{p}}+\frac{I\left(t\right)}{C_{p}} \end{array}\right.$$

where *U*_p_ is the voltage across the RC network. After discretization, Equation 1 can be expressed as:

(Equation 2) $$\left\{ \begin{array}{l} U_{p,k}=e^{-\frac{\Delta T}{\tau_{k-1}}}U_{p,k-1}+\left(1-e^{-\frac{\Delta T}{\tau_{k-1}}}\right)I_{k-1}R_{p,k-1}\\ U_{t,k}=U_{\text{OC},k}-I_{k}R_{0,k}-U_{p,k} \end{array}\right.$$

where, *τ* is the time constant and can be expressed as *τ* = *R*_p_*C*_p_, and Δ*T* is the time interval, which is set to 1 s in this work.

The state vector, input vector and output vector are defined as **x** = [*U*_OC_, *τ*, *R*_p_, *R*_0_, *U*_p_]^T^, **u** = *I* and **y** = *U*_t_, respectively. Considering that the changes in model parameters between adjacent time steps are small, the following assumptions can be made^32^:

(Equation 3) $$U_{\text{OC},k}\approx U_{\text{OC},k-1},\tau_{k}\approx \tau_{k-1},R_{p,k}\approx R_{p,k-1},R_{0,k}\approx R_{0,k-1}$$

Therefore, the following process equation can be obtained:

(Equation 4) $$f\left(x_{k-1},u_{k-1}\right)=\left[\begin{array}{l} U_{\text{OC},k-1}\\ \tau_{k-1}\\ R_{p,k-1}\\ R_{0,k-1}\\ e^{-\frac{\Delta T}{\tau_{k-1}}}U_{p,k-1}+\left(1-e^{-\frac{\Delta T}{\tau_{k-1}}}\right)I_{k-1}R_{p,k-1} \end{array}\right]$$

The measurement equation can be expressed as:

(Equation 5) $$g\left(x_{k-1},u_{k-1}\right)=U_{\text{OC},k}-I_{k}R_{0,k}-U_{p,k}$$

Based on Equations 4 and 5, the state space expression of the Thevenin model can be given by:

(Equation 6) $$\left\{ \begin{array}{l} x_{k}=f\left(x_{k-1},u_{k-1}\right)+\omega_{k}\\ y_{k}=g\left(x_{k},u_{k}\right)+\upsilon_{k} \end{array}\right.$$

where, **ω** is process noise and **υ** is measurement noise. According to Equations 4 and 5, the state transition matrix and the measurement matrix can be computed as:

(Equation 7) $$A_{k-1}={\frac{\partial f\left(x_{k-1},u_{k-1}\right)}{\partial x_{k-1}}|}_{x_{k-1}={\hat{x}}_{k-1}}=\left[\begin{array}{ccccc} 1 & 0 & 0 & 0 & 0\\ 0 & 1 & 0 & 0 & 0\\ 0 & 0 & 1 & 0 & 0\\ 0 & 0 & 0 & 1 & 0\\ 0 & \alpha & \beta & 0 & e^{-\frac{\Delta T}{{\hat{\tau }}_{k-1}}} \end{array}\right]$$

(Equation 8) $$\alpha =\frac{\Delta T\left({\hat{U}}_{p,k-1}-I_{k-1}{\hat{R}}_{p,k-1}\right)e^{-\frac{\Delta T}{{\hat{\tau }}_{k-1}}}}{{\hat{\tau }}_{k-1}^{2}}$$

(Equation 9) $$\beta =I_{k-1}\left(1-e^{-\frac{\Delta T}{{\hat{\tau }}_{k-1}}}\right)$$

(Equation 10) $$C_{k}={\frac{\partial g\left(x_{k},u_{k}\right)}{\partial x_{k}}|}_{x_{k}={\hat{x}}_{k}}=\left[\begin{array}{ccccc} 1 & 0 & 0 & -I_{k} & -1 \end{array}\right]$$

where the superscript “ˆ” represents the estimated value. Finally, the EKF is used for online identification of model parameters, which calculation process is shown in Table S3.

#### Rule I

Rule I assesses the accuracy of the identified OCV from two perspectives: the input signal and the identification result. The current signal must contain sufficient fluctuations and last long enough to ensure accurate identification of each parameter. It is essential to make real-time assessments of the operating conditions of EVs and to determine whether the input signals are valid. In this work, the SE is employed to evaluate the degree of current fluctuation, which can be calculated as^33^:

(Equation 11) $$\text{SE}=-\sum_{i=1}^{n}p_{i}\;\log_{c}p_{i}$$

where *p*_*i*_ is the probability density function, 0 ≤ *p*_*i*_ ≤ 1, $\sum_{i=1}^{n}p_{i}=1$. The base *c* determines the unit of the entropy. Commonly used bases include 2, e, and 10. In this work, e is adopted as the base of the logarithm. A one-dimensional SD is used to calculate the SE of the current, with a time interval of 1 s and a window size *w* of 60 s. The operating current range of the battery is divided into *n* equal parts. Specifically, the range of [-25 A–40 A] is divided into 13 subintervals, with a step size of 5 A. Then, the probability of each subinterval is represented by the number of samples falling in the subinterval:

(Equation 12) $$p_{i}=\frac{\#\left\{I_{j}|b_{i}\leq I_{j}<b_{i+1},I_{j}∍\text{SD}\right\}}{w},i=1,2,3,...,13$$

where, # represents the number of elements in the collection, and *b*_*i*_ is the boundary of the subinterval. Since the calculation of Equation 12 requires traversing the current in the SD, which is time-consuming, an iterative method is proposed to compute the SE. Given that the size of the SD is constant, when a new current signal arrives, the earliest current signal in the SD is discarded. In this case, the subinterval where the number of samples changes can only be the one to which the new current or the earliest current in the SD belongs. Therefore, at each time step, the SE is updated by modifying only the probability of the subinterval where the number of samples changes. First, the current range is shifted to the positive region by adding an offset of 25 A, and the subintervals are labeled 1 to 13. A label vector is then initialized to store the label of each current in the SD, and a count vector is initialized to record the number of samples in each subinterval. At the *k*th time step, after collecting a new current signal, its label is calculated by:

(Equation 13) l(k)=⌊(I(k)+25)/5⌋

Then, the sample count of the subinterval corresponding to the new current is incremented by one, while the count associated with the earliest current in the SD is decremented by one. As a result, the SE can be iteratively updated by:

(Equation 14) $$\begin{array}{l} {\text{SE}}_{k}={\text{SE}}_{k-1}-D_{\text{old}}+D_{\text{new}}\\ D_{\text{old}}=-\frac{n_{s}}{w}\ln\frac{n_{s}}{w}-\frac{n_{e}}{w}\ln\frac{n_{e}}{w}\\ D_{\text{new}}=-\frac{n_{s}-1}{w}\ln\frac{n_{s}-1}{w}-\frac{n_{e}+1}{w}\ln\frac{n_{e}+1}{w} \end{array}$$

where *n*_s_ and *n*_e_ are the sample counts of the subintervals corresponding to the earliest current in the SD and the new current, respectively. If the new current and the earliest current in the SD belong to the same subinterval, the SE does not need to be updated. In this way, traversal is avoided at each time step, and only four logarithmic computations are required. The calculation complexity of the logarithmic function can be further reduced by using a lookup table. When the current is distributed across multiple subintervals, the SE becomes large, indicating strong current fluctuations. Therefore, the input signal is considered valid when the SE exceeds a predefined threshold, which is determined experimentally and set to 1 in this work.

In addition, once the parameters of the Thevenin model are obtained, the terminal voltage can be calculated using Equation 2, and the simulation error can be obtained by:

(Equation 15) $$U_{\text{err},k}=\left|U_{t,k}-{\hat{U}}_{t,k}\right|$$

Under dynamic operating conditions, the identification results may exhibit some instability. In this work, *U*_err_ is used to filter out the most accurate identified OCV for constructing the OCV-SOC curve. A predefined threshold for *U*_err_ is determined experimentally and set to 5 mV. Certainly, the ${\hat{U}}_{p}$ identified from EKF can be directly employed to calculate *U*_err_, but for online parameter identification algorithms that do not treat ${\hat{U}}_{p}$ as a parameter to be identified, it remains necessary to use Equation 2 for calculation. It is worth noting that when the model parameters diverge, the error of ${\hat{U}}_{p}$ gradually accumulates. However, once the model parameters reconverge, since exp(-Δ*T*/*τ*) is less than 1, ${\hat{U}}_{p}$ will gradually return to the normal range. Although this process may take some time, it can be accelerated by appropriately initializing the polarization voltage or constraining its magnitude.

Overall, the flowchart for rule I is presented in Figure S3. The accuracy of the identified OCV is thoroughly assessed by evaluating both the input signal and identification result using SE and *U*_err_. Combining these two metrics helps avoid situations where parameter errors cancel each other out, resulting in a very small *U*_err_. If the identified OCV is deemed accurate, it will be used for constructing the OCV-SOC curve and rule II; otherwise, it will be discarded.

#### Rule II

Rule II, as illustrated in Figure S4, is proposed in this work to ensure the accuracy of the SOC corresponding to the OCV. First, the base segment is established using the full charge condition, as shown in Case 1. Specifically, it is necessary to establish a full charge judgment strategy based on the battery charging protocol. The most commonly used protocols are constant current and constant current-constant voltage (including multi-stage charging schemes). Under these protocols, the full charge state can be determined by monitoring both current and voltage. When the battery is fully charged, the SOC is set to 100%. Some manufacturers may impose limits on the depth of charge and discharge; in such cases, the specified upper SOC limit can be treated as the full charge point. The terminal voltage after a rest period can be taken as the OCV at 100% SOC. Subsequent OCV and SOC can then be obtained through online parameter identification and Ah counting, thereby forming the base segment.

The accuracy of Ah counting is affected by the cumulative errors of the maximum available capacity and current signal.^34^ This work assumes that the maximum available capacity of the battery has been estimated by advanced SOH estimation methods.^35^^,^^36^ To limit other cumulative errors, the SOC is reset after a period of Ah counting, each time the vehicle is powered on, or when switching modes. Then, the identified OCV met the rule I is assessed whether it falls within the base segment, i.e., whether it is higher than the OCV at the last point of the base segment. If it does, SOC can be obtained via linear interpolation, as shown in Case 2. It then serves as the start point for subsequent SOC calculation through Ah counting, as shown in Case 3. If OCV does not fall within the base segment, accurate SOC cannot be guaranteed, and no further action is taken. When the conditions are met again, the same process continues until the OCV-SOC curve construction is complete, as shown in the last figure of Figure S4. To prevent excessive memory usage, historical data are not stored. Instead, only the data from the previous moment is saved.

When the OCV and SOC at the current moment meet both rule I and rule II, the data point is added to the OCV-SOC curve under construction. To balance the accuracy and simplicity of the constructed OCV-SOC curve, the SOC interval between adjacent construction points is constrained to between 2% and 4%.

### Quantification and statistical analysis

In this study, to quantitatively evaluate the performance of the proposed method in OCV–SOC curve construction and SOC estimation, the root-mean-square error (RMSE), mean absolute error (MAE) and maximum absolute error (MaxAE) are used as evaluation metrics, that is:

(Equation 16) $$\text{RMSE}=\sqrt{\frac{1}{n}\sum_{i=1}^{n}\left(x_{i}-{\hat{x}}_{i}\right)}$$

(Equation 17) $$\text{MAE}=\frac{1}{n}\sum_{i=1}^{n}\left|x_{i}-{\hat{x}}_{i}\right|$$

(Equation 18) $$\text{MaxAE}=\max\left|x_{i}-{\hat{x}}_{i}\right|$$

where *n* is the number of samples, *x* is the measured OCV or SOC, and $\hat{x}$ is the estimated OCV or SOC. The method simulations were performed using Python and run on an Intel (R) Core (TM) i7-12700H CPU @2.30 GHz, 16.0 GB RAM.
