# Supplementary material for: Entropy-driven online open circuit voltage identification for precise state estimation in lithium-ion batteries
Source: iScience. 2025 Aug 6;28(9):113290. doi: 10.1016/j.isci.2025.113290 (PMC12496173; doi:10.1016/j.isci.2025.113290)
Supplement: Document S1. Figures S1–S4 and Tables S1–S3 [file mmc1.pdf]

iScience, Volume *28*

Supplemental information

Entropy-driven online open circuit voltage identification for precise state estimation in lithium-ion batteries

Zhengyang Li, Cheng Chen, Ruixin Yang, Hailong Li, and Rui Xiong

SUPPLEMENTAL FIGURES

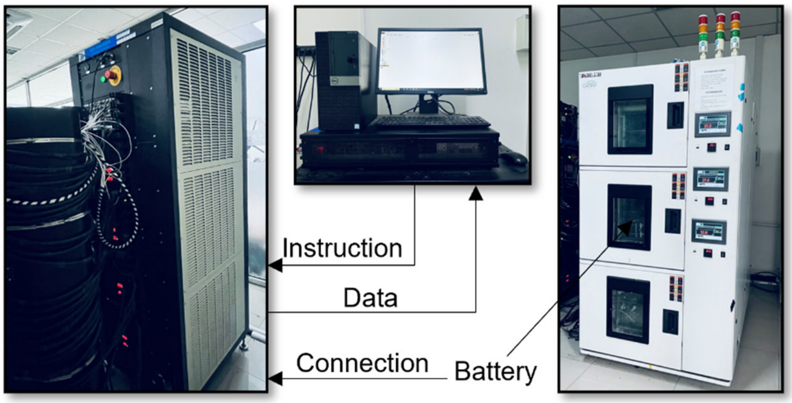

Figure S1. Experimental setup, related to STAR Methods.

A thermal chamber is used to provide the required environmental temperature for battery experiments. A battery test system is utilized to apply operating protocols to the battery and transmit information such as current, voltage, and temperature to a PC.

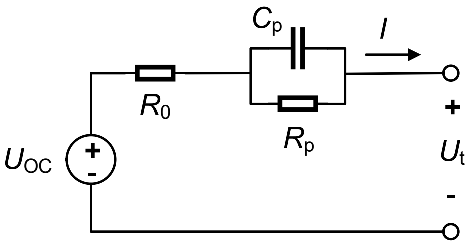

Figure S2. Thevenin model, related to STAR Methods.

The OCV is represented by a voltage source (U_{OC}). The ohmic resistance (R_0) stands for the sum of the resistances of current collectors, electrodes, and electrolyte. The parallel RC network (R_p and C_p) represents the polarization effect of the battery. U_t is the terminal voltage, and I is the current, with discharge being positive and charge being negative.

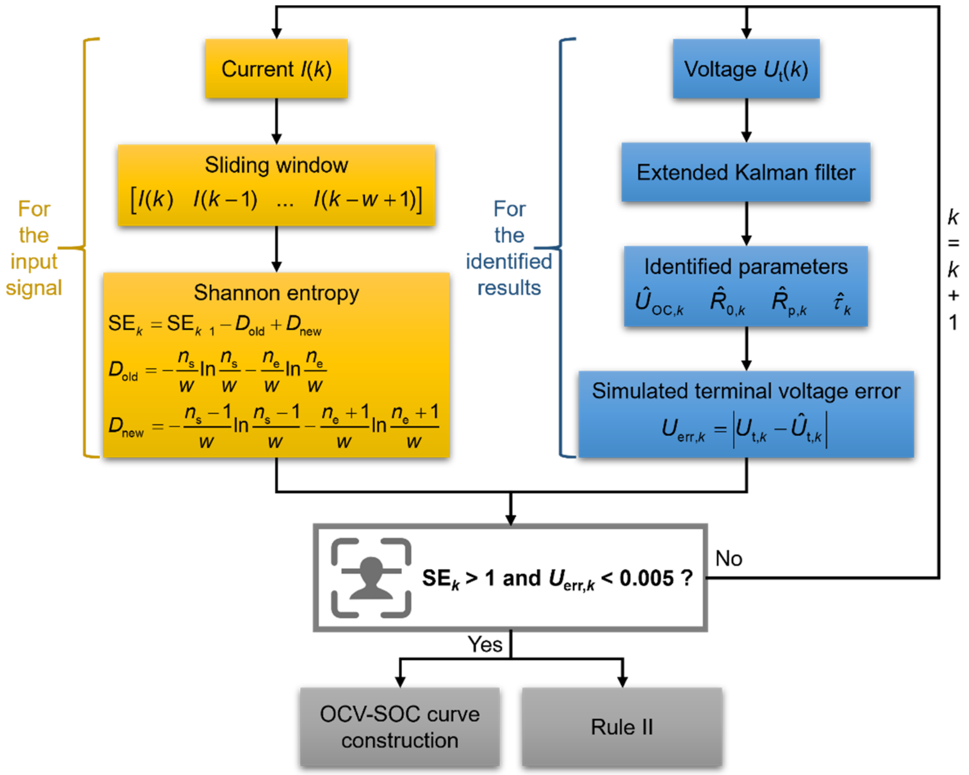

Figure S3. Flowchart of rule I, related to STAR Methods.

The accuracy of the identified OCV is thoroughly assessed by evaluating both the input signal and identification result using Shannon entropy (SE) and simulated terminal voltage error (U_{err}).

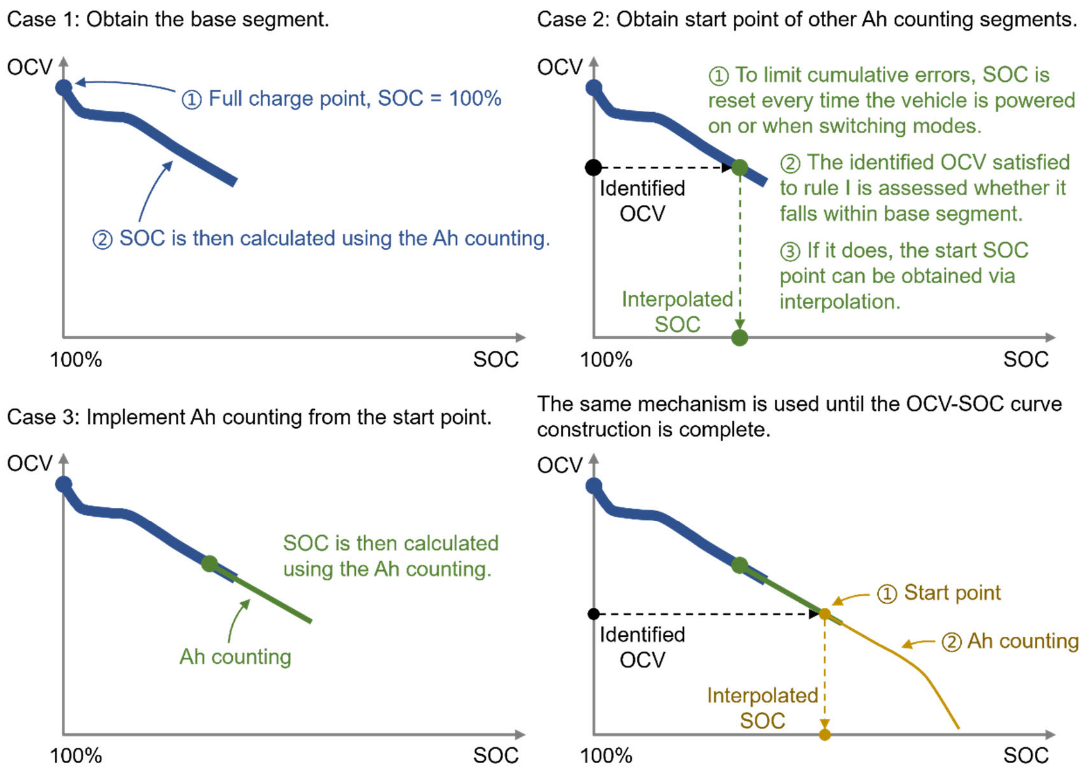

Figure S4. Mechanism of rule II, related to STAR Methods.

Start points are obtained via interpolation using the accurate OCV. SOC is then calculated using the Ah counting.

SUPPLEMENTAL TABLES

Table S1. Calculation process of SOC estimation based on the Thevenin model and EKF, related to Figure 10.

Step	Content
Initialization	$\hat{\mathbf{x}}_{z,0}^{+}=\left[\hat{U}_{\mathrm{p},0}\quad \hat{z}_0\right]^{\mathrm{T}}$,<br>$\mathbf{P}_z=\mathrm{diag}\left[1\quad 1\right]$,<br>$\mathbf{Q}_z=\mathrm{diag}\left[1\quad 1\right]\times 10^{-7}$,<br>$\mathbf{R}_z=10^{-1}$,<br>where the superscript "+" represents the posterior estimated value, $\mathbf{x}_z$ is the state vector, $\mathbf{P}_z$ is the covariance matrix of state estimation error, $\mathbf{Q}_z$ is the covariance matrix of process noise, and $\mathbf{R}_z$ is the covariance matrix of measurement noise.
Prior estimation	$\begin{cases}\hat{\mathbf{x}}_{z,k}^{-}=f_z(\hat{\mathbf{x}}_{z,k-1}^{+},\mathbf{u}_{k-1})\\ \mathbf{P}_{z,k}^{-}=\mathbf{A}_{z,k-1}\mathbf{P}_{z,k-1}^{+}\mathbf{A}_{z,k-1}^{\mathrm{T}}+\mathbf{Q}_z\end{cases}$,<br>where<br>$f_z(\mathbf{x}_{z,k-1},\mathbf{u}_{k-1})=\begin{bmatrix}\mathrm{e}^{-\frac{\Delta T}{\tau_{k-1}}}U_{\mathrm{p},k\text{-}1}+(1-\mathrm{e}^{-\frac{\Delta T}{\tau_{k-1}}})I_{k-1}R_{\mathrm{p},k\text{-}1}\\ z_{k-1}-\frac{I_{k-1}\Delta T\eta}{\mathrm{CAP}}\end{bmatrix}$,<br>$\mathbf{A}_{z,k-1}=\left.\frac{\partial f_z(\mathbf{x}_{z,k-1},\mathbf{u}_{k-1})}{\partial \mathbf{x}_{z,k-1}}\right\rvert_{\mathbf{x}_{z,k-1}=\hat{\mathbf{x}}_{z,k-1}}=\begin{bmatrix}\mathrm{e}^{-\frac{\Delta T}{\hat{\tau}_{k-1}}} & 0\\ 0 & 1\end{bmatrix}$,<br>the superscript "-" represents the prior estimated value, η is the charge-discharge efficiency, which is set to 1 in this work, and CAP is the battery capacity.
Kalman gain	$\mathbf{K}_{z,k}=\frac{\mathbf{P}_{z,k}^{-}\mathbf{C}_{z,k}^{\mathrm{T}}}{\mathbf{C}_{z,k}\mathbf{P}_{z,k}^{-}\mathbf{C}_{z,k}^{\mathrm{T}}+\mathbf{R}_z}$.
Posterior estimation	$\begin{cases}\hat{\mathbf{x}}_{z,k}^{+}=\hat{\mathbf{x}}_{z,k}^{-}+\mathbf{K}_{z,k}[\mathbf{y}_k-g(\hat{\mathbf{x}}_{z,k}^{-},\mathbf{u}_k)]\\ \mathbf{P}_{z,k}^{+}=(\mathbf{I}-\mathbf{K}_{z,k}\mathbf{C}_{z,k})\mathbf{P}_{z,k}^{-}\end{cases}$,<br>where $\mathbf{C}_{z,k}=\left.\frac{\partial g(\mathbf{x}_{z,k},\mathbf{u}_k)}{\partial \mathbf{x}_{z,k}}\right\rvert_{\mathbf{x}_{z,k}=\hat{\mathbf{x}}_{z,k}}=\begin{bmatrix}-1 & \left.\frac{\mathrm{d}U_{\mathrm{OC}}}{\mathrm{d}z}\right\rvert_{z=\hat{z}_k}\end{bmatrix}$, $\mathbf{I}$ is the identity matrix.

Table S2. Specifications of the tested LIB, related to STAR Methods.

Parameters	Value
Nominal capacity	30 Ah
Nominal voltage	3.62 V
Upper cut-off voltage	4.25 V
Lower cut-off voltage	3.0 V

Table S3. The calculation process of online parameter identification based on EKF, related to STAR Methods.

Step	Content
Initialization	$\hat{\mathbf{x}}_0^+ = \left[\hat{U}_{\mathrm{OC},0} \quad \hat{\tau}_0 \quad \hat{R}_{\mathrm{p},0} \quad \hat{R}_{0,0} \quad \hat{U}_{\mathrm{p},0}\right]^{\mathrm{T}}$,<br>$\mathbf{P}_0 = \mathrm{diag}[1 \quad 1 \quad 1 \quad 1 \quad 1]$,<br>$\mathbf{Q} = \mathrm{diag}[7 \quad 7 \quad 7 \quad 7 \quad 7] \times 10^{-8}$,<br>$\mathbf{R} = 10^{-2}$,<br>where the superscript "+" represents the posterior estimated value, **x** is the state vector, **P** is the covariance matrix of state estimation error, **Q** is the covariance matrix of process noise, and **R** is the covariance matrix of measurement noise.
Prior estimation	$\begin{cases} \hat{\mathbf{x}}_k^- = f(\hat{\mathbf{x}}_{k-1}^+, \mathbf{u}_{k-1}) \\ \mathbf{P}_k^- = \mathbf{A}_{k-1}\mathbf{P}_{k-1}^+\mathbf{A}_{k-1}^{\mathrm{T}} + \mathbf{Q} \end{cases}$,<br>where the superscript "-" represents the prior estimated value.
Kalman gain	$\mathbf{K}_k = \dfrac{\mathbf{P}_k^-\mathbf{C}_k^{\mathrm{T}}}{\mathbf{C}_k\mathbf{P}_k^-\mathbf{C}_k^{\mathrm{T}} + \mathbf{R}}$.
Posterior estimation	$\begin{cases} \hat{\mathbf{x}}_k^+ = \hat{\mathbf{x}}_k^- + \mathbf{K}_k[\mathbf{y}_k - g(\hat{\mathbf{x}}_k^-, \mathbf{u}_k)] \\ \mathbf{P}_k^+ = (\mathbf{I} - \mathbf{K}_k\mathbf{C}_k)\mathbf{P}_k^- \end{cases}$,<br>where **I** is the identity matrix.
